# The origins and longevity of IgE responses as indicated by serological and cellular studies in mice and humans

**DOI:** 10.1111/all.15799

**Published:** 2023-07-07

**Authors:** Zhoujie Ding, Jesse Mulder, Marcus J. Robinson

**Affiliations:** ^1^ Department of Immunology Monash University Melbourne Victoria Australia

**Keywords:** allergy, asthma, IgE, longevity, plasma cell

## Abstract

The existence of long‐lived IgE antibody‐secreting cells (ASC) is contentious, with the maintenance of sensitization by the continuous differentiation of short‐lived IgE^+^ ASC a possibility. Here, we review the epidemiological profile of IgE production, and give an overview of recent discoveries made on the mechanisms regulating IgE production from mouse models. Together, these data suggest that for most individuals, in most IgE‐associated diseases, IgE^+^ ASC are largely short‐lived cells. A subpopulation of IgE^+^ ASC in humans is likely to survive for tens of months, although due to autonomous IgE B cell receptor (BCR) signaling and antigen‐driven IgE^+^ ASC apoptosis, in general IgE^+^ ASC probably do not persist for the decades that other ASC are inferred to do. We also report on recently identified memory B cell transcriptional subtypes that are the likely source of IgE in ongoing responses, highlighting the probable importance of IL‐4Rα in their regulation. We suggest the field should look at dupilumab and other drugs that prohibit IgE^+^ ASC production as being effective treatments for IgE‐mediated aspects of disease in most individuals.

**Abbreviations**
ASC: antibody secreting cell(s)
BCR: B‐cell receptor
B‐lineage: B cells plus ASC
CSR: class‐switch recombination
GC: germinal center
iLC2: type 2 innate lymphoid cell
mBC: memory B cell
reg‐TFH: regulatory T follicular helper cell
SHM: somatic hypermutation
TFH: T follicular helper cell

## 1 INTRODUCTION

IgE is the rarest secreted isotype and antibody‐secreting cells (ASC) producing it are equally rare in abundance. Free IgE amounts can be regulated by dynamics of binding to cells carrying IgE receptors,^1^, ^2^ and also by the production of IgG anti‐IgE antibodies that can recognize free IgE.^3^, ^4^ Despite its rarity, IgE can reach high cell‐bound amounts and is a key mediator of allergy and associated diseases which impact health,^5^, ^6^, ^7^ starting with a process called “sensitization”. Sensitization relies on binding of allergen‐specific IgE to allergy effector mast cells and basophils, such that subsequent encounter with allergen triggers an allergic response. The response can range from mild tissue oedema, through gastrointestinal symptoms to potentially fatal anaphylaxis which most commonly occurs against ingested foods or administered drugs.^8^, ^9^, ^10^, ^11^, ^12^, ^13^, ^14^ Drugs such as BTK inhibitors limit IgE‐bound cell activation and can reduce severity of disease,^15^, ^16^ but despite six decades since the discovery of IgE,^17^ sensitization once established remains difficult to reverse. Current immunotherapy approaches seek to interrupt the production of IgE or to overwhelm IgE with high‐titer IgG to inhibit effector cell activation,^18^ with affinity and antibody amount influencing inhibition.^19^ Immunotherapy and monoclonal antibody treatment are often accompanied by allergic response elicitation and adverse events during treatment, for example, in oral food allergen desensitization^20^, ^21^ and in individuals treated with an anti‐IgE antibody (omalizumab) in asthma.^22^ However, interrupting IgE signaling can show persistent benefit, for example, in individuals treated for chronic idiopathic urticaria.^23^ Targeting IgE is generally beneficial in disease treatment^24^; in asthma, omalizumab treatment can reduce exacerbation rates,^24^, ^25^ while in IgE‐mediated allergies omalizumab and a second anti‐IgE antibody, ligelizumab, both reduce serum IgE amounts and show promise as adjuncts to immunotherapy^26^, ^27^; anti‐IgE treatment in the context of immunotherapy with allergen supports increases in allergen‐specific IgG4 production^28^ and increases the maximum tolerated dose in an oral food challenge.^29^ Omalizumab can further reduce the time taken to reach maintenance immunotherapy dose.^30^ Sustained nonresponsiveness, or tolerance is achieved with anti‐IgE plus immunotherapy in some individuals^31^; however, symptoms in many individuals return after cessation of the cotherapy,^32^ suggesting sustained nonresponsiveness, or tolerance mediated by T cells,^33^, ^34^ is not instated in many cases. Recently it has been acknowledged that glycosylation state of the IgE Fc region influences the interaction of omalizumab with free IgE,^35^ while glycosylation profiles of IgE antibodies differ by individual,^36^ so IgE glycosylation state might be relevant for sensitivity to such drugs. Understanding processes that perpetuate IgE production is necessary to stop reaginic activity in the long‐term.

Recent advances have clarified the origins of IgE responses, and therefore the means to reprogram toward tolerance. While there are still significant gaps in our understanding (Box 1), some notable discoveries have been made (Box 2). We know now that IgE^+^ ASC and B cells tend to evidence somatic hypermutation, suggesting a germinal centre (GC) or post‐GC memory B cell (mBC) source for the ASC, and can, but do not always share clonal overlap with IgG ASC.^37^, ^38^, ^39^, ^40^, ^41^ Similarly, in individuals with hyper IgE syndrome, IgE and IgG antibodies can bind to distinctive epitopes on allergens.^42^ Despite this, the memory of an IgE response resides primarily in the IgG class‐switched mBC pool.^43^, ^44^, ^45^ The identification of high IL‐4‐producing, IL‐13‐producing T follicular helper (TFH)13 cells may suggest a unique T‐helper cell that biases responses toward reaginic IgE production.^46^ Similarly, regulatory TFH (reg‐TFH) might contribute to allergen‐specific IgE production by producing IL‐10.^47^ By contrast, CTLA4, neuritin, and IL‐21 suppress IgE production.^48^, ^49^, ^50^ In addition to improving on scant data suggesting the existence of long‐lived IgE^+^ ASC, dissecting regulation of IgE^+^ ASC genesis from mBC and longevity of the ensuing response are notions that remain to be addressed. Here, we review cellular mechanisms by which IgE responses arise in mouse models, and what is indicated from recent clinical studies, attempting to put together typically disparate viewpoints on the origins of IgE in mouse models versus epidemiology.

BOX 1 **Major unknowns in cellular IgE response biology**

The transcriptional maturity of IgE^+^ ASC in bone marrow and intestinal tissues

The extent to which IgE^+^ ASC become long‐lived and their maximum potential life spans

Whether IgE class‐switch recombination is limited to the pre‐GC and memory phases, or commonly occurs within GC

Whether GC or post‐GC memory B cells provide for ongoing IgE^+^ ASC production in nonremitting allergies and mouse disease models

The ratio of allergen‐specific IgE^+^ B cells:mBC2 in peripheral blood of individuals with atopy

The identity of the CD4^+^ T cell that drives recall IgE responses

BOX 2 **Fundamental studies in IgE biology**

2007 IgE^+^ B‐lineage cells show an ASC bias, are mostly outside GC, but evidence SHM. Proposed that high‐affinity IgE arises from sequential IgG1 to IgE CSR^37^

2012 Proof of IgE^+^ GC B cell population and ASC differentiation predisposition^137^, ^138^

2017 IgG1^+^ B cells shown to hold IgE memory for recall responses in mice^43^

2018 Transcriptome of human IgE plasmablasts published, confirming SHM^38^

2019 General acceptance that IgE memory resides primarily among non‐IgE mBC in humans^147^, ^201^

2019 Discovery of TFH13 cells and regulation of IgE affinity by IL‐13^46^

2021 Neuritin discovered to suppress IgE responses^49^

2023 Type 2 memory B cells identified^44^, ^45^, ^144^

## 2 THE EPIDEMIOLOGY OF ALLERGY SUGGESTS CAREFUL CONSIDERATION OF THE ALLERGEN USED TO SENSITIZE IS REQUIRED IN MOUSE MODELS

**FIGURE 1:**
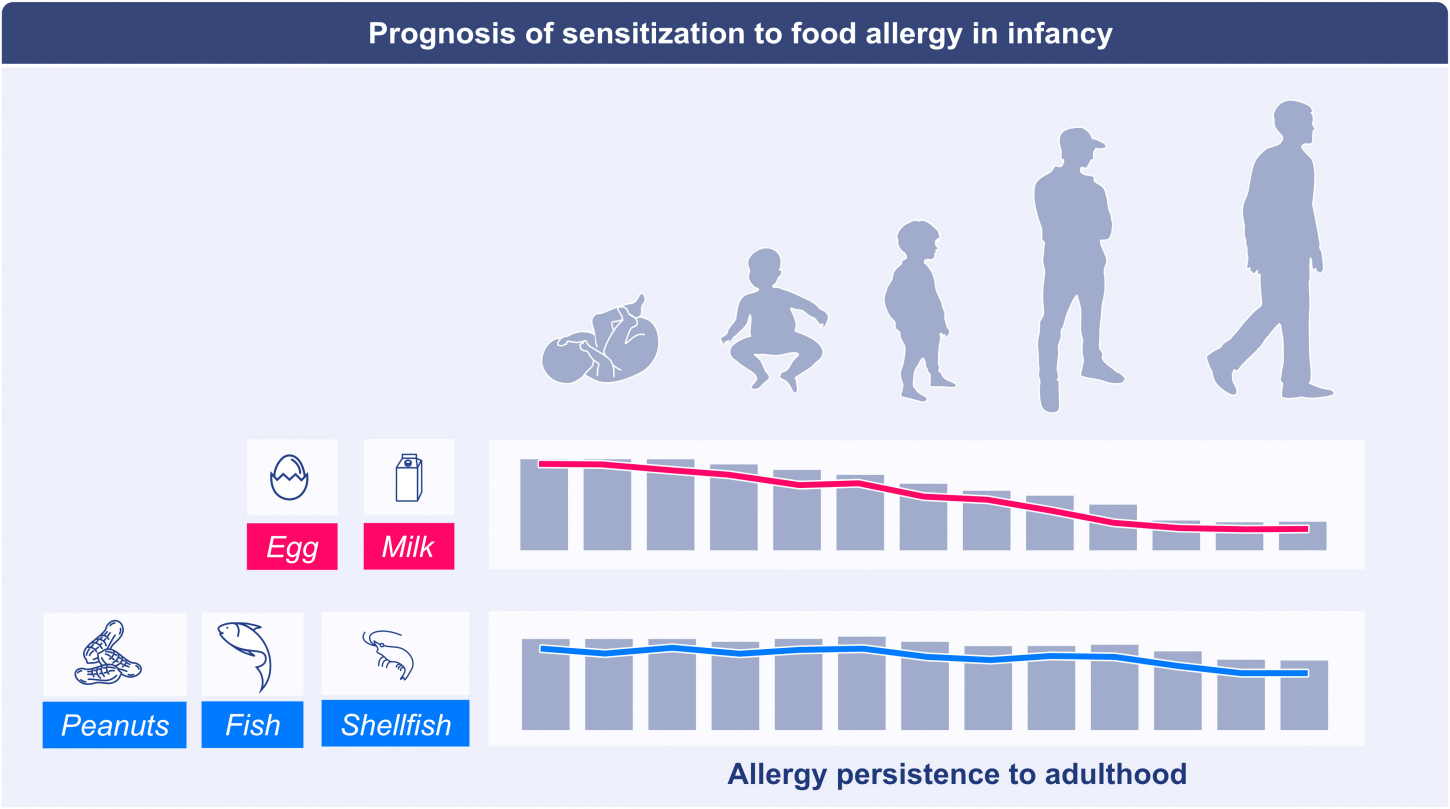
Prognosis of sensitization to food allergens in infancy. Food allergies have different rates of resolution as children age, with a majority of egg and milk allergies resolving by early adulthood; by contrast, allergies to peanuts, fish, and shellfish tend to be lifelong.

During the first 3 years of life, most allergies that develop are to food proteins, whereas the prevalence of environmental allergen sensitization increases after this time.^51^, ^52^ Interestingly, severe atopic dermatitis in the first year of life, but not thereafter, may predict likelihood of sensitization to environmental allergens as children age.^53^ Atopic dermatitis and asthma development are both associated with shifts in microbial colonization in the first year of life.^54^, ^55^ Further, food allergies and allergic rhinitis (to environmental allergens) have both seen an increase in incidence in recent decades.^51^, ^56^ Different foods have different likelihoods of sensitizing infants, and allergies to them have different resolution rates as children age (Figure 1).^57^, ^58^, ^59^ Similarly, environmental allergens stratify in likelihood of sensitization.^60^ For food allergy, although accurate determination of incidence is challenging and varies by geographic location, milk and egg allergies tend to predominate in infants,^57^, ^61^ although in countries with high consumption of fish, fish allergies can be quite prevalent in infants and children as well.^62^ The majority of milk and egg allergies resolve by age 12.^61^, ^63^, ^64^ By contrast, allergies to peanuts, tree nuts, fish, and shellfish, although less frequent, are unlikely to resolve.^64^, ^65^, ^66^ That is, there is something about certain allergens that affects the disease prognosis. Allergies likely to persist into adulthood are associated with higher amounts of serum IgE, with distinctive and diverse IgE‐binding epitope patterns on allergen, more diverse allergen recognition, and different V gene use of B cells relative to individuals that are tolerant to allergen or regularly ingesting it.^67^, ^68^, ^69^, ^70^, ^71^, ^72^, ^73^ An allergy to one protein is also likely to copresent with allergies to others.^28^, ^60^, ^74^, ^75^ For example, egg allergic individuals are more likely to develop a peanut allergy, and cockroach‐sensitized individuals are often cosensitized to house dust mite.^57^, ^60^ Thus, there are features of the allergens contained within certain substances that make them more likely to sensitize, in addition to a subset of individuals being prone to generating IgE responses. Unraveling allergy thus requires careful consideration of the substance used to generate the IgE response and of the host immune system. This means that haptens and model allergens such as ovalbumin are not appropriate substances for understanding the propagation of IgE responses against all allergens, at all times.

## 3 HOW DOES SENSITIZATION HAPPEN?

**FIGURE 2:**
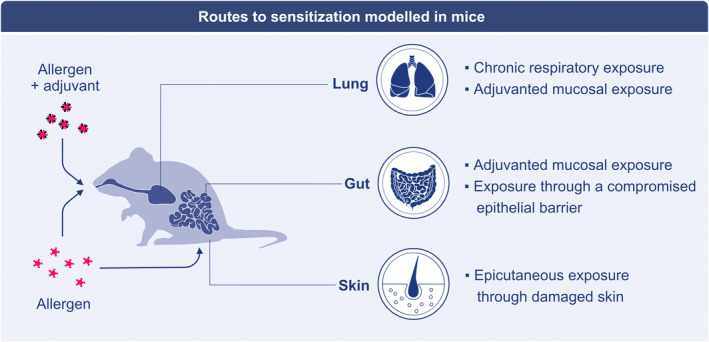
Routes to sensitization modeled in mice. Mouse models typically fall on the premise of (i) chronic respiratory exposure; (ii) adjuvanted mucosal exposure through the gut or lung; or (iii) exposure through tape‐strip damaged skin (epicutaneous exposure). Each route generates IgE, but the biology of the IgE response in each case may differ.

The manner in which allergen is first experienced has huge implications for sensitization outcome.^76^ Some individuals are prone to IgE production, a state termed atopy, as indicated by genome‐wide association studies identifying single nucleotide polymorphisms associating with IgE responses.^77^ However, various routes of sensitization may also contribute heterogeneity. The common routes of sensitization modeled in mice include chronic exposure, adjuvanted exposure, and exposure through a compromised epithelium (Figure 2).

### 3.1 Chronic exposure to allergens

While many allergies initially present in childhood, chronic workplace allergen exposure can result in adult sensitization, for example, as occurs in woodmill workers, fish packers, and bakers, the latter continually exposed to wheat flour,^78^, ^79^, ^80^ presumably via inhalational exposure. Healthcare workers that use latex gloves over long time periods are also at increased risk of developing latex allergies,^81^ presumably from exposure through the skin. We suggest that what we most closely approximate in mouse allergy models relying on long‐term repeated unadjuvanted allergen exposures, such as aerosolization of ovalbumin, are these chronic workplace exposures, where repeat challenge maintains a low‐level IgE response for long periods.^82^

### 3.2 Adjuvanted exposure to allergens

Another way that IgE responses develop is by adjuvanting the allergen exposure. Several models rely on this manner of driving IgE, for example, with low‐dose bacterial lipopolysaccharide,^83^ cholera toxin,^84^ or alum^85^‐based sensitizations. Although such models could be seen as nonphysiological, recently we suggested they might be relevant to human sensitization, as there are pathogens associated with each of the dominant food allergens to which individuals become sensitized in early life, and pattern recognition receptor ligands might thus be coexposed with the allergen at some point.^86^ In seafood processing factories, detectable levels of endotoxins are found in the air, which might contribute to sensitization by workplace allergens.^87^ It was also recently suggested that, as many allergens carry small molecules that are immunogenic, such as lipopolysaccharide, it is possible that these small molecules contribute to sensitization.^88^ We speculate that adjuvanted exposures mimic these states, wherein components from the infectious agent or small molecule act as adjuvants to enhance sensitization against the innocuous substance in which the molecule was harbored. This may occur under conditions that are sufficient to stimulate alarmin (IL‐25, IL‐33, and TSLP) production and activate an immune response, perhaps by damaging or activating epithelial cells, without triggering overt IFNγ production from resident myeloid cells, avoiding Th1 biasing of the immune response, so we would suggest a possible scenario where it is weakly immunogenic pathogen‐associated molecular pattern exposures that contributes to this form of sensitization, as has been indicated previously.^89^ Other allergens, such as papain, Der p 1, and Der p 2 have auto‐adjuvanting properties due to their proteolytic activity, molecular mimicry of important innate immune pathway coreceptors such as MD2 or ability to activate pattern recognition receptors.^90^, ^91^, ^92^, ^93^, ^94^ Birch pollen allergen extracts may even directly interact with the IL‐4Rα to promote sensitization.^95^ In these cases, the basis of allergy might be understood by considering the adjuvanting properties of substances as contributors to sensitization.

### 3.3 Tape‐stripping and damaged epithelia

A systemic IgE priming may also occur by exposing tape‐strip damaged epidermis to allergen,^96^ with the exposure to allergens through eczematous skin lesions or a damaged epithelial barrier, such as occurs with filaggrin loss‐of‐function mutations that render individuals prone to atopic diseases,^97^, ^98^ potentially underlying sensitization, setting the epitopes that are recognized by IgE in allergic individuals.^71^ Damage to epithelial barriers caused by detergents and environmental pollutants is a tenet of the “epithelial barrier” hypothesis, seen as a major cause of multiple diseases, including those mediated by type 2 immunity and associated with IgE.^99^, ^100^, ^101^ A recently identified subset of dendritic cells responsive to IL‐13 that promotes type 2 immunity may be key to IgE production after allergen exposure through a compromised epithelial barrier,^102^ but alarmins, such as IL‐33 likely also play a role.^91^, ^96^ Similarly, a compromised intestinal epithelial barrier may also permit sensitization to otherwise well tolerated dietary proteins.^103^ In summary, models of allergy reflect three separate sensitization routes: chronic exposure; adjuvanted exposure through a mucosal route; and exposure through compromised epithelial barriers (Figure 2).

## 4 THE REGULATION OF IGE PRODUCTION BY T CELLS AND IL‐4R SIGNALING

**FIGURE 3:**
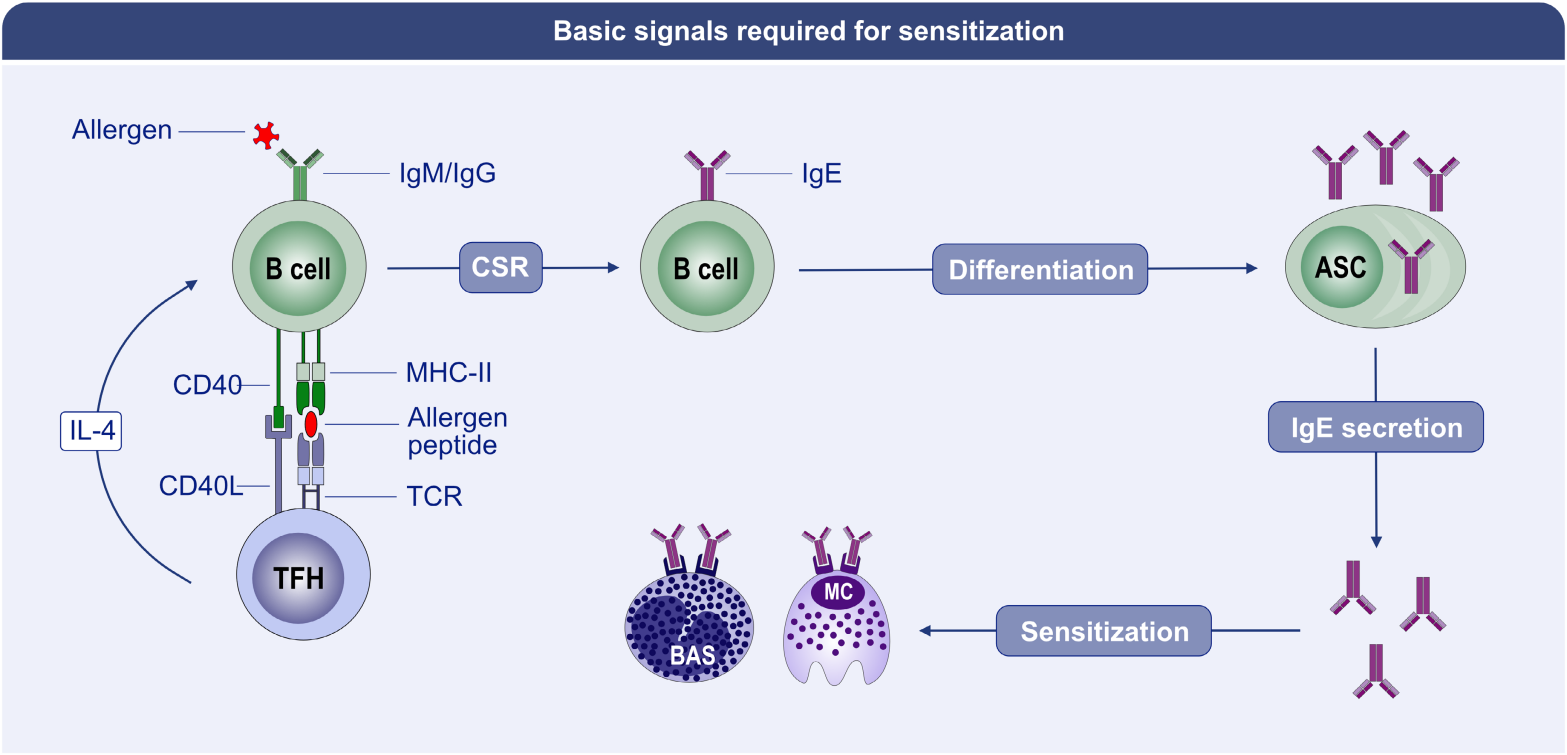
Basic signals required for sensitization. Upon initial allergen exposure, B cells capture antigen through their B cell receptors (BCR), and process and present peptides from that allergen via MHC‐II to cognate T follicular helper (TFH) cells, which recognize peptide–MHC‐II complexes through the T cell receptor. The T cell provides the cognate B cell with IL‐4 and costimulation via CD40L (signaling through CD40 on the B cell) to drive B cell proliferation and IgE class‐switch recombination (CSR). Once a B cell is IgE class‐switched, it can then differentiate into an IgE antibody‐secreting cell (ASC), which produces IgE to bind to high‐affinity IgE receptors (FcϵRI) on the surface of mast cells and basophils, resulting in sensitization.

Sensitization requires a set of basic signals (Figure 3). The most important stimulus for IgE class‐switch recombination (CSR) is signaling via IL‐4Rα and STAT6, with IL‐4 being a potent IgE‐genic cytokine in both primary and recall responses.^104^, ^105^, ^106^, ^107^ A key role for CD40 signaling and CD4^+^ T cell help is known for initiating IgE CSR.^50^, ^108^, ^109^ Mouse studies show that lymphoid IL‐4 derives predominantly from CD4^+^CXCR5^+^PD‐1^hi^ TFH cells,^110^ and IgE production requires BCL6‐expressing T cell‐intrinsic IL‐4 production,^111^, ^112^ that is, IL‐4 production from TFH rather than GATA‐3‐dependent Th2 cells, which mediate type 2 immunity in the tissues.^113^ Note that T cell‐intrinsic, BCL6‐dependent IgE production does not restrict the IgE response to GC‐sourced T cell help, because T cells express BCL6 before GC form, as well as outside GC in human lymph nodes.^114^, ^115^, ^116^ Furthermore, it is necessary to acknowledge that basophils can contribute prodigious amounts of IL‐4 in primary and secondary responses,^117^, ^118^ so in contexts of initial or allergen re‐exposure, IL‐4 may also derive systemically from basophils. Similarly, GATA3^+^ Lineage^negative^ type 2 innate lymphoid cells (iLC2), while typically known for their role as effector cells producing IL‐5 and IL‐13 in tissues in type 2 immune responses including atopic dermatititis and allergic asthma,^119^, ^120^, ^121^ can be stimulated with phorbol myristate acetate and ionomycin to produce IL‐4 when isolated from lymph nodes,^122^ and further contribute to elevating serum IL‐4 amounts after helminth infection.^123^ Notably, IL‐13^+^ high IL‐4‐producing, TFH13 cells were recently reported to promote the production of high‐affinity IgE in mice and are found specifically in allergic individuals.^46^ However, the conditions under which TFH13 cells arise also result in low TFH cell IL‐21 production.^46^ As IL‐21 tends to suppress IgE production,^50^ hence it could also be that the balance between IL‐4 and IL‐21 regulates the amount of IgE CSR that occurs in GC. While this indicates that GC‐localized TFH13 provide the help required for reaginic IgE production, it remains possible that extra‐GC TFH contribute to IgE^+^ ASC formation and potentially in responses elicited by allergen re‐exposures.

## 5 CELLULAR SOURCES OF IGE ^+^ ASC

IgE B‐lineage (B cells plus ASC) cells have been known for some time to be inherently ASC‐biased,^37^ but the cellular states from which IgE^+^ ASC arise are a matter of ongoing study; however, strong indications suggest that GC or post‐GC mBC commonly give rise to IgE^+^ ASC. GC are the sites in which B cells undergo somatic hypermutation (SHM), selection for expansion, and ASC differentiation, ultimately resulting in affinity maturation of serum antibody.^124^ GC are also shown to be the overwhelming source of long‐lived ASC in mice.^125^, ^126^ Several studies have analyzed the mutational burdens of IgE^+^ ASC, finding multiple BCR mutations in many of the identified IgE^+^ ASC, suggesting a common GC or post‐GC mBC origin.^39^, ^127^, ^128^ Reaginic IgE typically is of high affinity in mice, also suggesting a GC source.^46^ Sequential switching via IgG1 in mice was proposed^37^ and has since been shown to be the main mechanism by which such IgE arises,^129^ although, a recent study challenges this notion.^130^ Interestingly, GC B cells express IL‐13Rα1,^46^ and BCL6‐driven IL‐13 deletion reduces IgE antibody affinity to nonreaginic thresholds, indicating the capacity of GC B cells to respond to IL‐13 may influence IgE affinity.^46^ IgE responses also ensue via the extrafollicular route for long periods in parallel with GC responses,^131^ and unmutated clones are found among IgE^+^ B‐lineage cells of atopic individuals.^39^ Therefore, during type 2 immune responses such as allergies, IgE^+^ ASC can arise via the extrafollicular route, but mainly form from the differentiation of GC or post‐GC mBC populations; whether GC or post‐GC mBC are the dominant source is unclear.

### 5.1 Switching to IgE in primary responses

**FIGURE 4:**
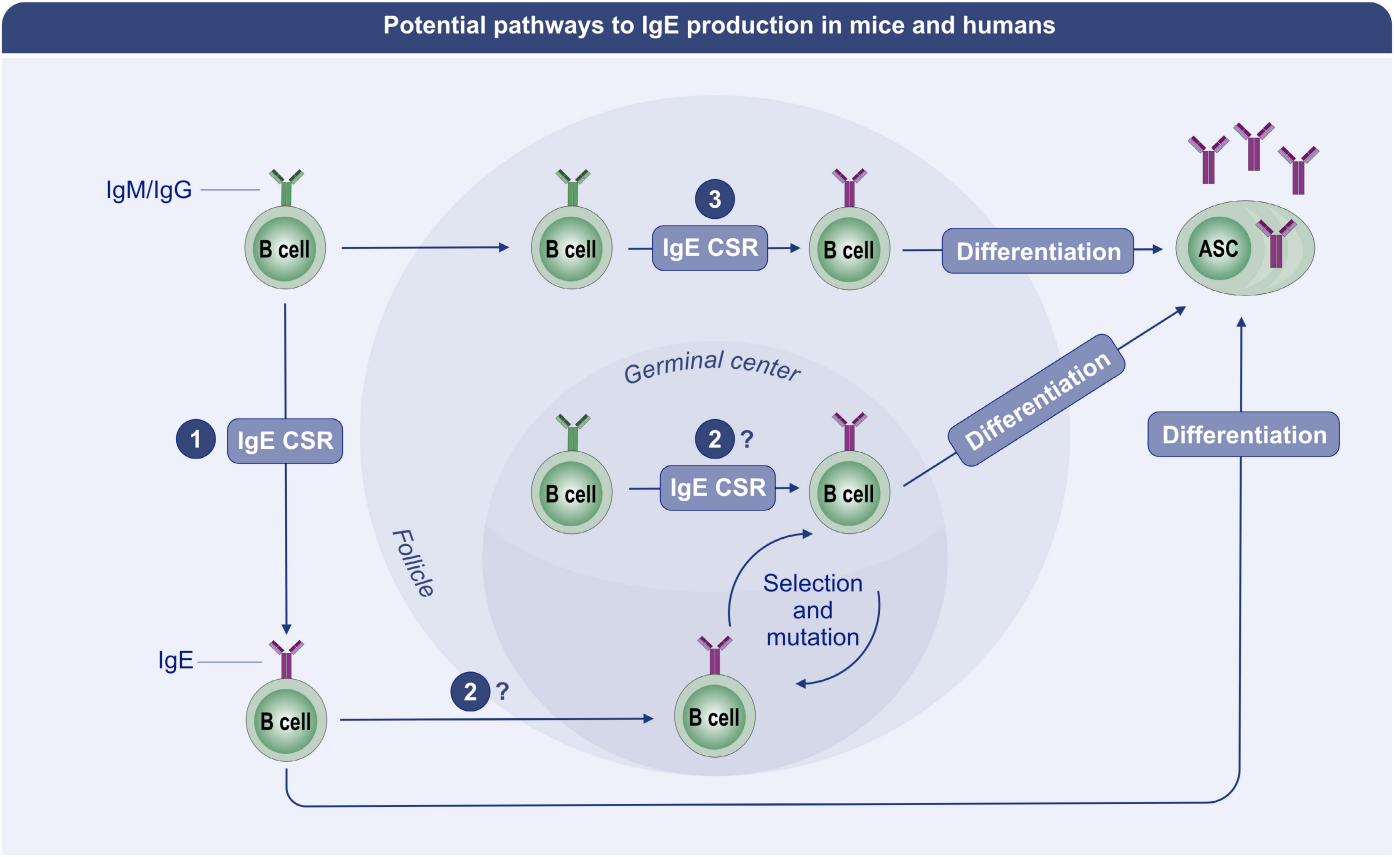
Potential pathways to IgE production in mice and humans. IgE class‐switch recombination (CSR) may occur during (1) the preGC phase of a B cell response, to give rise to IgE^+^ preGC B cells, which go on to become short‐lived extrafollicular IgE^+^ ASC. They also (2) likely enter GC to generate IgE^+^ B cells. Alternatively, IgM/IgG^+^ GC B cells may switch to IgE within GC to allow IgE^+^ GC B cells to then become GC‐derived IgE^+^ ASC. Which is the case has not been definitively demonstrated. (3) IgM/IgG^+^ memory B cells that can arise both directly from the preGC pool and via GC differentiation, can switch to IgE to generate IgE^+^ ASC upon allergen re‐exposure. Post‐GC IgE^+^ ASC have been convincingly demonstrated by BCR mutational profiles, but whether they arise directly from IgE^+^ GC B cells, via an IgM/IgG^+^ GC switch intermediate, or predominantly from switching at the memory B cell stage is not clear.

Before GC form, there is a period in which activated B cells seek out T cell help at the follicle borders.^124^ During this pre‐GC phase, the localization of B cells is controlled by expression of the chemokine receptor EBI2.^132^ Intrinsic silencing of EBI2 is required for mouse pre‐GC B cell relocalization to the follicle centre to form GC,^132^ which is mediated by the upregulation of BCL6, a master transcription factor for GC responses.^133^ BCL6 silences Iϵ expression in B cells, precluding IgE CSR,^134^ which is rare in GC in general and most shown to occur during the preGC phase,^135^ although IgE CSR was not specifically evaluated in that study. However, from this premise, IgE CSR should be more likely during the pre‐GC period in which B cells upregulate BCL6 to low amounts,^136^ rather than the high amounts expressed in committed GC B cells.^136^ It has further been shown that IgE^+^ B cells are lost from GC with time.^137^, ^138^ Still, as IgE^+^ ASC with heavily mutated BCR may arise in chronic allergen exposure models,^139^ it would be warranted to study IgE^+^ B cell persistence in GC throughout such a response. This is especially important as IgE^+^ and IgG1^+^ B‐lineage cells display overlap in clonality, but certain clones can be preferentially expanded among IgE^+^ B‐lineage cells, which also tend to have lower SHM burdens than contemporaneously isolated IgG^+^ B‐lineage cells,^37^, ^39^, ^40^, ^43^, ^129^, ^131^, ^137^ suggesting that there are overlapping, yet distinct processes regulating IgE and IgG production. ASC can arise from differentiation directly from a GC B cell, or via the reactivation of mBC near the subcapsular sinus.^140^, ^141^ Thus, IgE^+^ preGC B cells likely go on to become GC B cells, but further IgE^+^ GC B cells may be generated during the response from local switching; alternatively IgE^+^ B cells may be mainly generated from switching at the memory stage (Figure 4). Which is the dominant formation route of IgE ASC is a current question. It may be possible to visualize this with in vivo microscopy in dual fluorescent IgE and ASC reporter mouse strains, and literally see whether GC^137^ or the recently defined subcapsular sinus proliferative foci^141^ are the sites of IgE^+^ ASC differentiation, but sequencing studies also provide insight into IgE^+^ ASC relationships to precursors.^39^, ^131^

### 5.2 IgE ^+^ ASC formation upon allergen re‐exposure

Historical data suggest that B cells of an isotype other than IgE mediate recall IgE responses.^142^ Mouse work suggests that IgG B cells can be the main reservoir of IgE^+^ ASC precursors in challenge contexts.^43^, ^143^ Interestingly, two recent human studies,^44^, ^45^ and two new preprints [discussed in^144^], identify IgG^+^ mBC transcribing germline ϵ transcript, *IL4RA* and *IL13RA1*, suggesting these are cells poised to switch to IgE but reliant on further exposure to IL‐4 or potentially IL‐13 to make that switch.^44^, ^45^ In ex vivo culture with IL‐4 and CD40 stimulation, atopic individuals with high numbers of such cells show enhanced IgE production relative to healthy controls with fewer of them.^45^ The IgG cells uniquely express the gene encoding CD23,^45^ the low‐affinity IgE receptor, potentially indicating a feedback role for IgE in regulating antigen recognition by these cells when in proximity to allergen‐specific IgE‐secreting ASC. Going forward, it will be interesting to learn the abundance of these “type 2 mBC”^144^ in cases of immunotherapy with concomitant IL‐4Rα inhibition to establish whether type 2 mBC maintenance is an IL‐4/IL‐13 dependent process. It will also be important to unravel in mouse models the relative importance of GC B cells versus type 2 mBC in propagating IgE responses upon allergen re‐exposure.

In contrast to IgG^+^ B cells harboring IgE memory, one group identified IgE^+^ B cells with PCR‐amplified Sμ‐Sε switch regions, suggesting that rare IgE^+^ mBC exist.^145^, ^146^ However, without transcriptomic confirmation, most flow cytometrically identified IgE^+^ cells are false positives,^9^, ^38^, ^147^ typically caused by cytophilic antibody binding, antibody bound via CD23, cross‐reactivity of detection reagents or the incomplete removal of doublet cell events and cellular debris, and it is suggested IgE^+^ mBC are so rare as to be irrelevant for IgE production in most allergic individuals.^9^, ^38^ False detection in “IgE^+^” fractions is a significant problem that requires staining of other isotypes and careful subsetting to avoid in multiple IgE reporter mouse strains as well.^138^, ^143^, ^148^ Given the predisposition of IgE^+^ B cells to differentiate into ASC in an antigen‐independent manner,^137^, ^149^, ^150^, ^151^, ^152^ it might indicate that rare IgE^+^ “memory” B cells are rather in a transient state on the way to ASC differentiation,^149^, ^150^, ^151^, ^152^ or perhaps apoptosis.^151^, ^153^ Resolving if this is the case will require transcriptomics on peripheral blood IgE^+^ B cells to see if they carry hallmarks of quiescence, proliferation or differentiation. Equally, to formally conclude these cells are irrelevant for IgE response propagation will require analysis of their abundance relative to type 2 mBC, to determine the order of magnitude by which type 2 mBC outnumber IgE^+^ mBC as a putative IgE source in different disease states, such as hyper‐IgE, chronic idiopathic urticaria, and allergy.

One of the nuances of IgE memory is that recall responses are CD4^+^ T cell and IL‐4 dependent just like in the primary response.^106^, ^108^, ^154^ A specific CD4^+^ T cell that supports secondary IgE^+^ ASC differentiation has yet to be defined, although a derivative of TFH13 cells seems a likely candidate. However, there is nothing transcriptionally “unique” about the subcapsular sinus foci T cells that are interacting with ASC precursors in secondary responses relative to those in secondary GC,^155^ meaning that work needs to be done to define the source of help for IgE production in challenge contexts.

### 5.3 Suppression of IgE ^+^ ASC production by neuritin, CTLA4, and IL‐21

**FIGURE 5:**
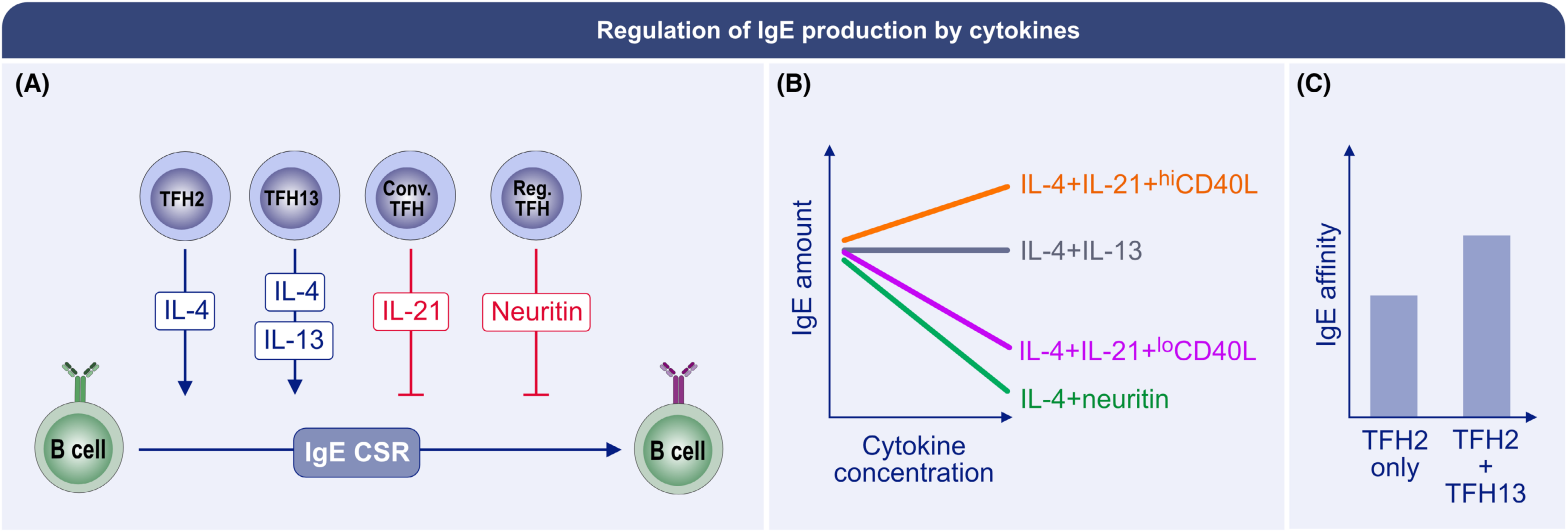
Regulation of IgE production by cytokines. (A) Newly identified TFH13 cells^46^ as well as TFH2 cells produce IL‐4 that promotes IgE class‐switch recombination. It is not clear if this most commonly occurs primarily prior to GC formation, once GC are established, or during recall responses in subcapsular sinus foci. By contrast, conventional TFH (conv‐TFH) and reg‐TFH produce high amounts of IL‐21 and neuritin, respectively, to suppress IgE CSR and therefore IgE^+^ ASC formation.^49^ (B) In culture, addition of neuritin to activated B cells diminishes IgE amounts in a concentration‐dependent manner.^49^ IL‐21 can suppress IgE production, or enhance it, depending on the amount of CD40 stimulation the B cells receive.^50^ (C) While IL‐13 has minimal effect on IgE amounts in murine B cell culture, in vivo its absence limits the affinity maturation of IgE antibodies such that they are not reaginic when it is genetically deleted from TFH cells, and when IL‐13‐expressing TFH are deleted.^46^

In contrast to IL‐4, neuritin, produced by reg‐TFH, was recently shown to inhibit mouse and human IgE production in vitro and play a role in IgE regulation in vivo by acting directly on murine B cells^49^ (Figure 5). CTLA4 is similarly expressed by reg‐TFH and also inhibits IgE production.^48^, ^156^, ^157^ In mice, IL‐21 has pleiotropic effects on B cells,^158^, ^159^ including being a major constrainer of IgE production.^50^ Cultures of human B cells have suggested IgE production is enhanced by IL‐21,^160^ but a comprehensive recent study^50^ plus an earlier work together indicate that the inhibitory capacity of IL‐21 on IgE production in cultured B cells is overridden by strong CD40 signaling,^50^, ^161^ reconciling the findings (Figure 5). Interestingly, the recent study^50^ showed that much of the suppressive capacity of IL‐21 on IgE^+^ ASC formation in mice occurs through STAT3, as would be predicted from human work which shows IL‐21R loss‐of‐function mutations as a cause of hyper‐IgE in ≈40% of such individuals, with defective phosphorylation of STAT3 and heightened Th2 cytokine production being probable underlying causes.^162^ The study further showed that deletion of IFNγ, once considered a suppressor of the IgE response,^106^ made no difference to IgE^+^ ASC abundances, indicating impacts of IFNγ on IgE production are indirect.^50^

## 6 THE LIFE SPANS OF IGE ^+^ ASC

Recent clinical findings confirm the importance of IL‐4 in the ongoing production of IgE. Individuals with high IgE at baseline in several diseases show clinical benefit with anti‐IL‐4Rα (dupilumab) treatment, for example, in alopecia areata, allergic asthma, and atopic dermatitis.^163^, ^164^, ^165^, ^166^ Dupilumab causes downmodulation and internalization of IL4Rα^167^ to interfere with the IL‐4 signaling that is an absolute requirement for IgE CSR.^106^ While dupilumab might affect additional aspects of atopic disease,^168^, ^169^, ^170^ dupilumab leads to a 20%–50% drop in serum IgE over several months,^166^, ^171^, ^172^, ^173^, ^174^ and >80% loss over treatment periods of 1 year or more^175^, ^176^, ^177^, ^178^ in allergic asthma, allergic rhinitis with or without nasal polyps, and atopic dermatitis patients, which fits with the notion that most IgE^+^ ASC are short‐lived cells in these diseases. Dupilumab also diminishes peanut‐specific IgE titres >90% over a 3‐year timecourse.^178^ Two studies suggest limited antigen‐specific IgE titer change in pollen allergic individuals over a four‐month period with dupilumab alone, which would instead indicate persistent IgE^+^ ASC cause pollen allergy.^179^, ^180^ Intriguingly, in one of those studies, patients concomitantly undergoing subcutaneous immunotherapy had IgE titres that dropped 50%–60%,^179^ arguing putatively persistent IgE^+^ ASC were being killed by exposure to cognate antigen, as would be predicted from a recent mouse study where IgE BCR ligation was shown to cause IgE^+^ ASC apoptosis.^181^ Additional human studies point to a long‐lived IgE^+^ ASC population by showing IgE titer maintenance in periods of allergen avoidance,^182^ but it is possible that without IL‐4R blockade IgE^+^ ASC production occurs constantly from GC or mBC. A study of dupilumab treatment in atopic dermatitis patients argues that the rare patients whose IgE levels are unchanged might be those in which IL‐4R signaling is not completely blocked.^183^ A maintained IgE titer is also inconsistent with long‐lived ASC decaying at an intrinsic rate, as we and others recently demonstrated occurs for long‐lived ASC in mice,^125^, ^184^, ^185^ which might support that production was ongoing.

### 6.1 Evidence for short‐lived IgE ^+^ ASC

**FIGURE 6:**
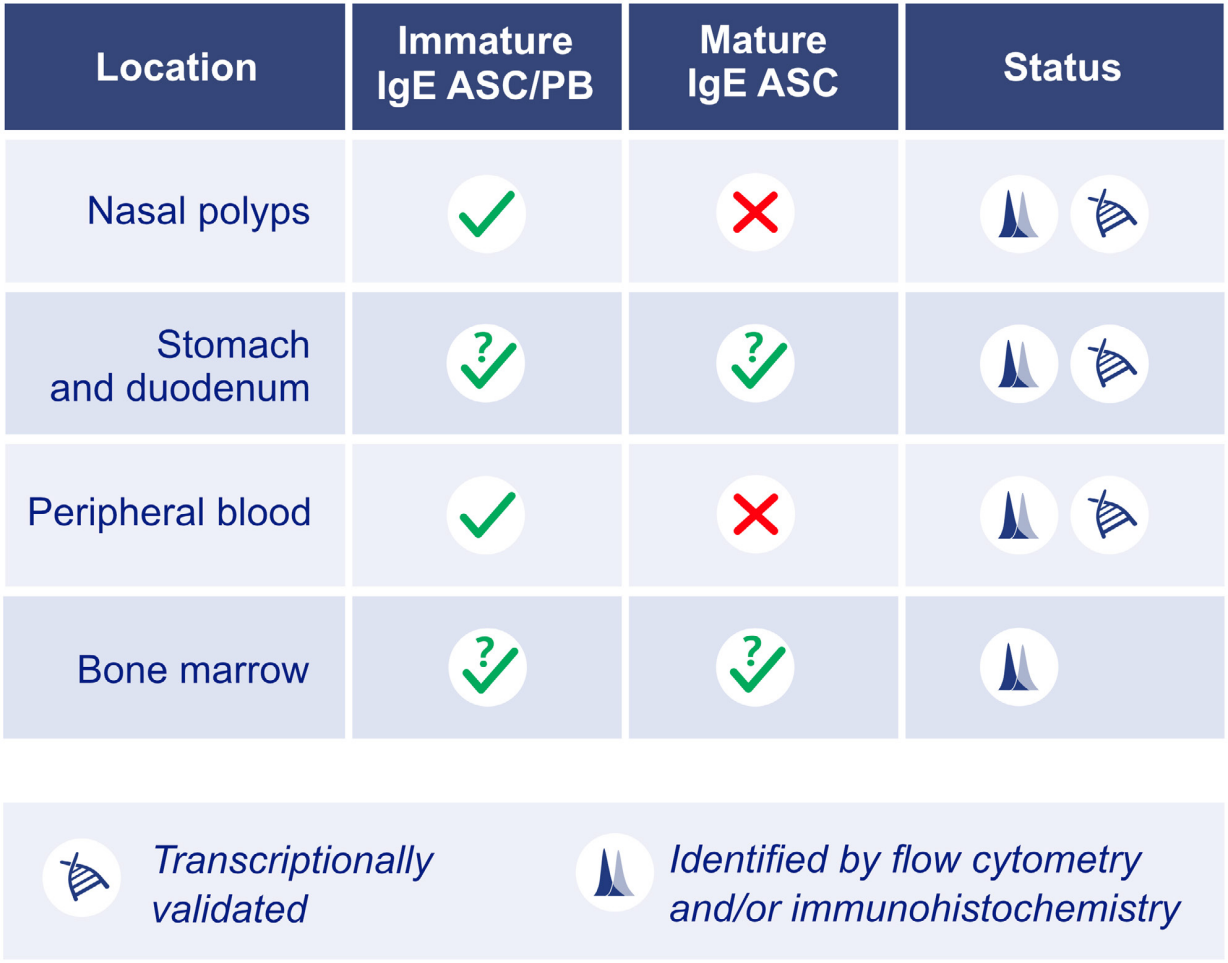
Sites of residence of IgE^+^ ASC. IgE^+^ ASC have been identified transcriptomically in nasal polyps, peripheral blood, and stomach and duodenal sections from humans.^38^, ^39^, ^127^, ^189^, ^190^, ^191^ In the case of blood and nasal polyp‐localized IgE^+^ ASC, these were defined as transcriptionally immature or plasmablasts.^38^, ^191^ IgE^+^ ASC have been found in bone marrow by flow cytometry and immunohistochemistry, but their transcriptomic state has not been resolved as plasmablast, immature, or mature ASC.^139^, ^194^ Similarly, in the intestine, it is not clear if IgE^+^ ASC are plasmablasts, immature, or mature ASC, despite convincing evidence they can reside at that site.^127^, ^190^

IgE^+^ ASC have been identified in several tissues by flow cytometry, most notably, in peripheral blood and in nasal polyps, which is associated with local IgE production and detection of target antigens in the tissue.^38^, ^128^, ^186^, ^187^, ^188^ Transcriptomically validated IgE^+^ ASC have been detected in peripheral blood of individuals with atopic diseases,^38^, ^39^ in dissected polyps from individuals with allergic rhinitis with nasal polyps, and intestinal mucosa of peanut allergic individuals^127^, ^189^, ^190^, ^191^ (Figure 6). Transcriptome analysis of IgE^+^ ASC suggest that the bulk resolvable IgE^+^ ASC are plasmablasts or immature ASC, rather than mature plasma cells, as they maintain expression of MHC‐II, a marker that is down‐modulated in the first few days after ASC obtain quiescence.^38^, ^125^, ^191^ Interestingly, one study showed that IgE^+^ ASC lost expression of *MKI67*, a marker of the proliferative plasmablast state, suggesting that IgE^+^ ASC can become quiescent.^191^ Thus, while transcriptomic evidence has confirmed that immature IgE^+^ ASC exist, we await transcriptomic depictions of mature IgE^+^ ASC or bone marrow IgE^+^ ASC with potential for longevity (Figure 6). Mechanistically, the IgE BCR undergoes autonomous antigen‐independent signaling^151^, ^152^, ^153^ with BCR stimulation of IgE^+^ ASC leading to apoptosis,^181^ and it has further been proposed that IgE^+^ ASC do not become long‐lived because they are prone to death via a BCL2L11 dependent pathway,^192^ which may suggest that IgE^+^ ASC are intrinsically disposed to being short‐lived and therefore continually produced.

### 6.2 Do long‐lived IgE ^+^ ASC exist?

IgE^+^ ASC have been found residing in bone marrow, potentially indicating a long‐lived IgE^+^ ASC population exists under some circumstances.^139^, ^193^, ^194^ A caveat to this is that we recently showed most bone marrow ASC are short‐lived in mice,^125^ showing that residence there does not guarantee longevity.

Some indication that IgE^+^ ASC can persist is evident in a model wherein mice are ovalbumin‐sensitized and then exposed to cyclophosphamide. Seventy days later, ovalbumin‐specific IgE^+^ ASC are detected in spleen and bone marrow by ELISpot.^195^ This suggests that the ovalbumin‐specific IgE^+^ ASC were persistent cells, as cyclophosphamide should prohibit new ASC production. However, the degree to which cyclophosphamide blocked production was not demonstrated. Similarly, in a model of house dust mite allergy, venus IgE‐reporter positive ASC were detected in bone marrow samples 9 weeks after house dust mite exposures were stopped.^139^ Anti‐CD20 treatment, which eliminates B cells, and putatively prevents ongoing IgE^+^ ASC formation made no difference to the rate of decline of serum IgE in the model over a 32‐week period.^139^ One limitation of the study is that anti‐CD20 treatment can deplete peripheral blood B cells without depleting GC B cells.^196^ Hence, without confirming GC ablation, it is not certain that IgE^+^ ASC production was altered. A second limitation in the study is that the venus^+^ cells were not evaluated for expression of other Ig isotypes—in IgE reporter mice, cells expressing other isotypes can comprise high proportions of reporter positive cells, and a proportion of IgG1^+^ ASC express IgE reporter fluorescence in the strain used to track reporter positive ASC in bone marrow.^137^, ^138^, ^139^, ^143^, ^148^ As discussed earlier, false positives in IgE^+^ cell fractions requires careful subsetting to avoid.^148^ If “IgE^+^” cells are not significantly less abundant than IgG^+^ cells, loosely reflecting the serum antibody differential (100‐2000‐fold lower for IgE than IgG^81^), probably there is contamination in the gate. Further, IgE^+^ ASC show a CXCL12 homing defect, placing them at a disadvantage at seeding in bone marrow, which may suggest ASC in other tissues could equally contribute to ongoing IgE production.^153^, ^197^ Genetic time stamping of ASC in mice is now possible,^126^, ^184^, ^185^ so calculating the life span of IgE^+^ ASC by such a method is probably the next step toward assessing their life span potential.

**FIGURE 7:**
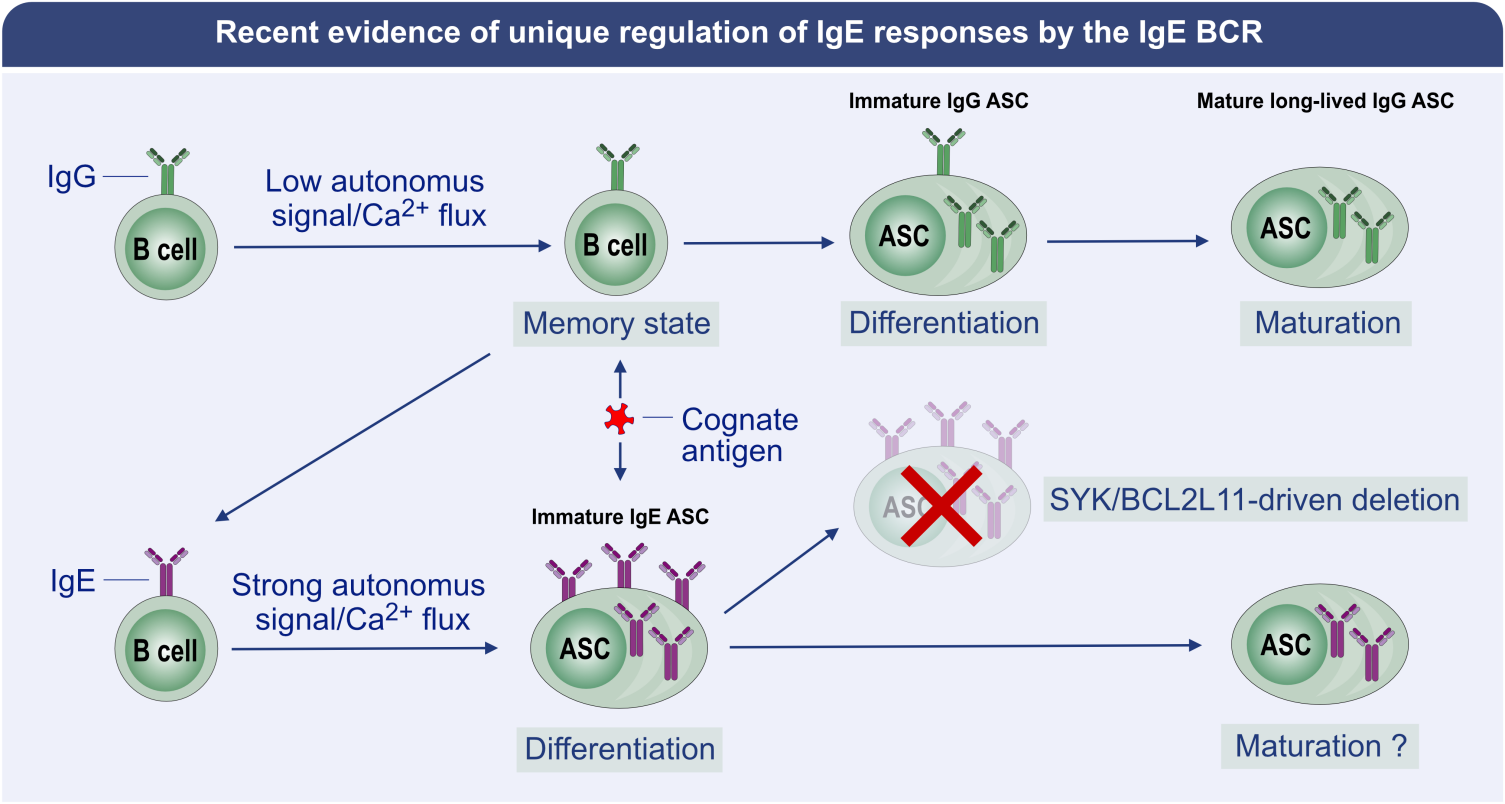
Recent evidence of unique regulation of IgE responses by the IgE BCR. gG1^+^ B cells in mice exhibit limited autonomous signaling,^151^, ^152^ and are shown to enter quiescence as memory B cells. Upon antigen ligation of the IgG1 BCR, IgG1^+^ B cells differentiate into IgG1^+^ ASC,^151^, ^152^ which can migrate to bone marrow and mature into long‐lived IgG1^+^ ASC.^126^ In contrast, IgE^+^ B cells exhibit strong autonomous BCR signaling, which is associated with a predisposition to differentiation into ASC.^149^, ^151^, ^152^ Whether IgE^+^ ASC can transition to maturity and remain as long‐lived in bone marrow is unclear; however, antigen ligation of the IgE BCR on ASC promotes SYK‐ and BCL2L11‐dependent apoptosis,^181^, ^192^ limiting the life span of most IgE^+^ ASC.

Life span tracing of IgE^+^ ASC is also especially relevant because ASC survival is heterogeneous, dispersed across a continuum, rather than being cells in a fixed short‐lived or long‐lived state.^125^, ^185^ Human data confirm that splitting ASC into two discrete populations is a false dichotomisation: serum titer decline reveals tetanus and diphtheria specific ASC to have half‐lives of approximately 10 years, whereas those recognizing smallpox or human papilloma virus antigens persist with immortal half‐lives.^198^, ^199^ The life spans of ASC induced by some vaccines can average 7–11 months, for example, for specificities such as SARS‐CoV‐2 spike protein^188^ while other vaccines such as influenza split‐virus vaccines fail to generate populations of ASC that survive for even that length of time.^199^ In line with such stratifications, dupilumab treatment of one patient over several years suggested that IgE titres reached extremely low levels, such that the “long‐lived” IgE^+^ ASC were inferred to have a life span limited to tens of months.^200^ From these data, we speculate that IgE^+^ ASC of different specificities might stratify, influencing the etiology of disease—to explain the discordant fates of infants sensitized to egg versus peanut, it might be that peanut‐specific IgE^+^ ASC survive for longer periods than those recognizing egg do. If persisting IgE^+^ ASC are eventually identified, given the revelations of the autonomous signaling of the IgE BCR predisposing IgE^+^ B cells to ASC differentiation and the predisposition of IgE^+^ ASC to apoptosis with BCR ligation^151^, ^152^, ^181^ (Figure 7), it is unlikely that they survive indefinitely, and might be amenable to removal by BCR‐mediated killing.^181^

## 7 CONCLUSIONS

Recent discoveries have uncovered the unique biology of IgE responses. These include that a population of high IL‐4‐producing, low IL‐21‐producing TFH13 cells mediates reaginic IgE production,^46^ that expression of the IgE BCR provides an intrinsic predisposition toward ASC differentiation^151^, ^152^, ^153^ and potentially apoptosis^151^, ^153^, ^181^, ^192^ and that mouse and human IgG class‐switched, post‐GC mBC are the likely reservoir of IgE^+^ ASC forming cells in re‐exposure contexts.^43^, ^44^, ^45^ A more contentious finding is the persistence of IgE^+^ ASC in bone marrow.^139^ In the coming years it will be necessary to resolve the biology of IgE^+^ ASC generated by (i) chronic allergen exposure; (ii) adjuvanted, mucosal allergen exposure; and (iii) through epicutaneous sensitization. Further, studying the transcriptome of the rare IgE^+^ ASC located in human bone marrow will be telling as to whether IgE^+^ ASC can attain a long‐lived transcriptional state. This will position the field to understand the processes that underlie sensitization in diverse contexts and will be key to diminishing IgE^+^ ASC production and survival in the long‐term.

## GLOSSARY

Germinal center—the anatomical site in lymphoid tissues in which B cells undergo somatic hypermutation, with those possessing higher affinity for antigen actively selected by CD4^+^  T cells to become antibody‐secreting cells; Reagin—IgE antibody that facilitates anaphylaxis; Sensitization—the production of allergen‐specific IgE and its binding to mast cells and basophils sufficient to trigger an allergic response upon allergen re‐exposure; TFH2 cell—a follicular T helper cell that produces primarily IL‐4 and low IL‐21 amounts; TFH13 cell—a follicular T helper cell that produces high IL‐4 amounts, IL‐13 and low IL‐21 amounts; Tolerance—the lack of a deleterious immune response against a substance, such that if a food allergen, it can be incorporated into the diet without harmful effects. Type 2 memory B cell (mBC2)—an IL‐4Rα^+^CD23^+^B cell that also transcribes germline ϵ transcript and has been through an immune response, considered ‘poised’ to become an IgE antibody‐secreting cell upon allergen re‐exposure.

## AUTHOR CONTRIBUTIONS

MJR was involved in conceptualization, writing—original draft, funding acquisition and supervision. JM and ZD were involved in writing—review and editing. MJR, JM, and ZD were involved in visualization.

## CONFLICT OF INTEREST STATEMENT

The authors declare no financial conflicts of interest pertaining to this review.

## Data Availability

Data sharing is not applicable to this article as no new data were created or analyzed in this study.
